# Modulation of Cytokine Secretion Allows CD4 T Cells Secreting IL-10 and IL-17 to Simultaneously Participate in Maintaining Tolerance and Immunity

**DOI:** 10.1371/journal.pone.0145788

**Published:** 2015-12-28

**Authors:** Kanako Saito, Pascale Pignon, Maha Ayyoub, Danila Valmori

**Affiliations:** 1 Institut National de la Santé et de la Recherche Médicale, Unité 1102, Equipe Labellisée Ligue Contre le Cancer, Institut de Cancérologie de l’Ouest, Nantes-Saint Herblain, France; 2 Faculty of Medicine, University of Nantes, Nantes, France; McGill University Health Center, CANADA

## Abstract

CD4 T cells secreting IL-10 or IL-17 are frequent at mucosal sites, where their equilibrium is important for simultaneously maintaining tolerance and immunity to the resident microbiota. The mode of action of these cells, however, is as yet incompletely understood. In this study, we have combined *ex vivo* analysis of CD4 T cells producing IL-10 or/and IL-17 with assessment of clonal populations isolated *ex vivo* using a cytokine catch assay. We found that circulating CD4 T cells secreting IL-10 or/and IL-17 *ex vivo* include both conventional FOXP3^-^ CD4 T cells and FOXP3^+^ Helios^-^ Treg. Upon assessment of clonal populations derived from single *ex vivo* isolated cytokine secreting cells, we found that IL-10 or/and IL-17 secreting cells prevalently secrete one or the other cytokine depending on the type of stimulation, the time after stimulation and the presence of microbial products. Namely, IL-10 secretion by clonal cells was prevalent at early time points after TCR mediated stimulation, was independent of co-stimulation and was increased in the presence of the microbial fermentation product butyrate. In contrast, IL-17 secretion was higher at later time points after TCR mediated stimulation and in the presence of co-stimulatory signals. Taken together, these results provide insights into the mechanisms that, through modulation of cytokine secretion depending on conditions, allow IL-10 and IL-17 producing CD4 T cells to contribute to maintain tolerance to microbes locally, while retaining the ability to participate in protective immune responses at distant sites.

## Introduction

CD4 T cells can play dual and potentially opposing roles in immune responses, acting either as helper/effector (T_H_) or regulatory/suppressor T cells (Treg). CD4 T cells secreting IL-10 have been predominantly described as immunosuppressive and include both Tr1 cells and FOXP3^+^ Treg [1–3]. In contrast, CD4 T cells secreting IL-17 have been mostly described as helper/effectors (T_H_17). T_H_17 have been extensively studied because of their implication in autoimmunity but they have also been shown to participate in host defense against fungi, extracellular bacteria and cancer [4, 5]. An increasing body of experimental evidence, however, indicates that some CD4 T cells secreting IL-17 can be of regulatory type, as human FOXP3^+^ Treg that secrete IL-17 *ex vivo* while concomitantly exerting suppressive activity have been described by us and others [4, 6, 7]. Also, murine T_H_17 cells induced by TGF-β have been shown to suppress T cell immunity via the action of CD39 and CD73 ectonucleotidases [8]. CD4 T cell populations secreting IL-17 or IL-10 are frequent in the gut, where their equilibrium is important for simultaneously maintaining tolerance and immunity to the resident microbiota [9]. Although T_H_17 and IL-10-secreting CD4 T cells have mostly been considered as distinct populations, the generation of which involves distinct factors [10], Staphylococcus Aureus specific T_H_17 clones that co-secrete the two cytokines have been recently identified [11] underlining the close relationship between the two populations, along with their relevance in host immunity. The mode of action of human CD4 T cell populations secreting IL-10 or/and IL-17, however, is as yet incompletely understood. To get insight into these questions, in this study, we have combined *ex vivo* analysis of CD4 T cells producing IL-10 or/and IL-17 with assessment of clonal populations isolated *ex vivo* using a cytokine catch assay. We found that circulating CD4 T cells secreting IL-10 or/and IL-17 *ex vivo* include both conventional FOXP3^-^ CD4 T cell populations and FOXP3^+^ Helios^-^ peripherally induced Treg (pTreg). Upon assessment of clonal populations derived from single *ex vivo* isolated cytokine secreting cells, we found that IL-10 or/and IL-17 secreting cells prevalently secrete one or the other cytokine depending on the type of stimulation, the time after stimulation and the presence of local factors. Based on these results, we hypothesize that the ability of CD4 T cells co-producing pro- and anti-inflammatory cytokines to modulate their cytokine production profile depending on a combination of factors, might allow them to simultaneously contribute to maintain tolerance to commensal microbiota in their normal setting while retaining the ability to participate in immune responses against them when they are present at other sites.

## Materials and Methods

### Samples and cell purification

Peripheral blood samples were obtained from the Etablissement Français du Sang Pays de la Loire (Nantes, France) upon informed consent and were approved by the Institutional Review Board of the Etablissement Français du Sang Pays de la Loire (Nantes, France). A signed informed consent form was obtained anonymously from all healthy donors participating in the study. PBMCs were isolated by density gradient sedimentation using LSM 1077 lymphocyte separation medium (PAA Laboratories). CD4^+^ T cells were enriched by positive selection from PBMCs by magnetic cell sorting (Miltenyi Biotec).

### *Ex vivo* assessment of cytokine production and expression of transcription factors

For the assessment of cytokine secreting populations and transcription factor expression, CD4^+^ T cells were stimulated with PMA (100 ng/ml; Sigma Aldrich) and ionomycin (1 μg/ml; Sigma Aldrich) during 6hrs, and brefeldin A (10 μg/ml; Sigma Aldrich) was added 1hr after the beginning of the incubation. Cells were then stained with anti-CD45RA (BD Bioscience) Abs, where specified, fixed, permeabilized using the Foxp3 Staining Buffer Set (eBioscience) according to the manufacturer’s instructions, stained with Abs specific for IL-2 (BD Bioscience), IL-10 (BD Bioscience), IL-17 (eBioscience), IFN-γ (BD Bioscience), FOXP3 (eBioscience) and Helios (BioLegend), as indicated, and analyzed by flow cytometry (FACSAria II; BD Biosciences).

### *Ex vivo* isolation of cytokine secreting cells and generation of clones

CD4 T cells secreting IL-10 or/and IL-17 *ex vivo* were isolated using a two-color catch assay combining the IL-17-APC and IL-10-PE secretion assays from Miltenyi Biotec. Briefly, cells were stimulated with PMA/ionomycin during 5hrs, washed and incubated with the catch reagents and with the detection reagents according to the manufacturer instructions. Cytokine secreting populations were separated by flow cytometry cell sorting (FACSAria II; BD Biosciences). Isolated populations were cultured under limiting dilution conditions in the presence of PHA (1 μg/ml; Sigma Aldrich), recombinant human IL-2 (150 U/ml; Chiron) and irradiated feeder cells. Clones were harvested 10 to 15 days later and expanded under the same conditions in 96-well U bottom plates.

### Assessment of cytokine secretion by isolated clones

To assess cytokine secretion, clones were stimulated with plate-bound anti-CD3 (2 μg/ml; eBioscience) or/and -CD28 (6 μg/ml; eBioscience) Abs or with PMA and/or ionomycin, as indicated. Cytokines were measured in the 24hrs culture supernatants, by ELISA (IL-10; Life technologies, IL-17; R&D systems). Where indicated, cells were stimulated in the presence of sodium butyrate (0.1 mM; Sigma-Aldrich).

### Statistical analysis

Statistical analyses were performed with GraphPad Prism 6 (GraphPad Software Inc., San Diego, CA, USA). Groups were compared using unpaired Student’s *t*-test. In the cases where statistically significant differences were found P values were presented in the figure legends.

## Results

### *Ex vivo* secretion of IL-10 and IL-17 in human circulating CD4 memory T cells

**Fig 1:**
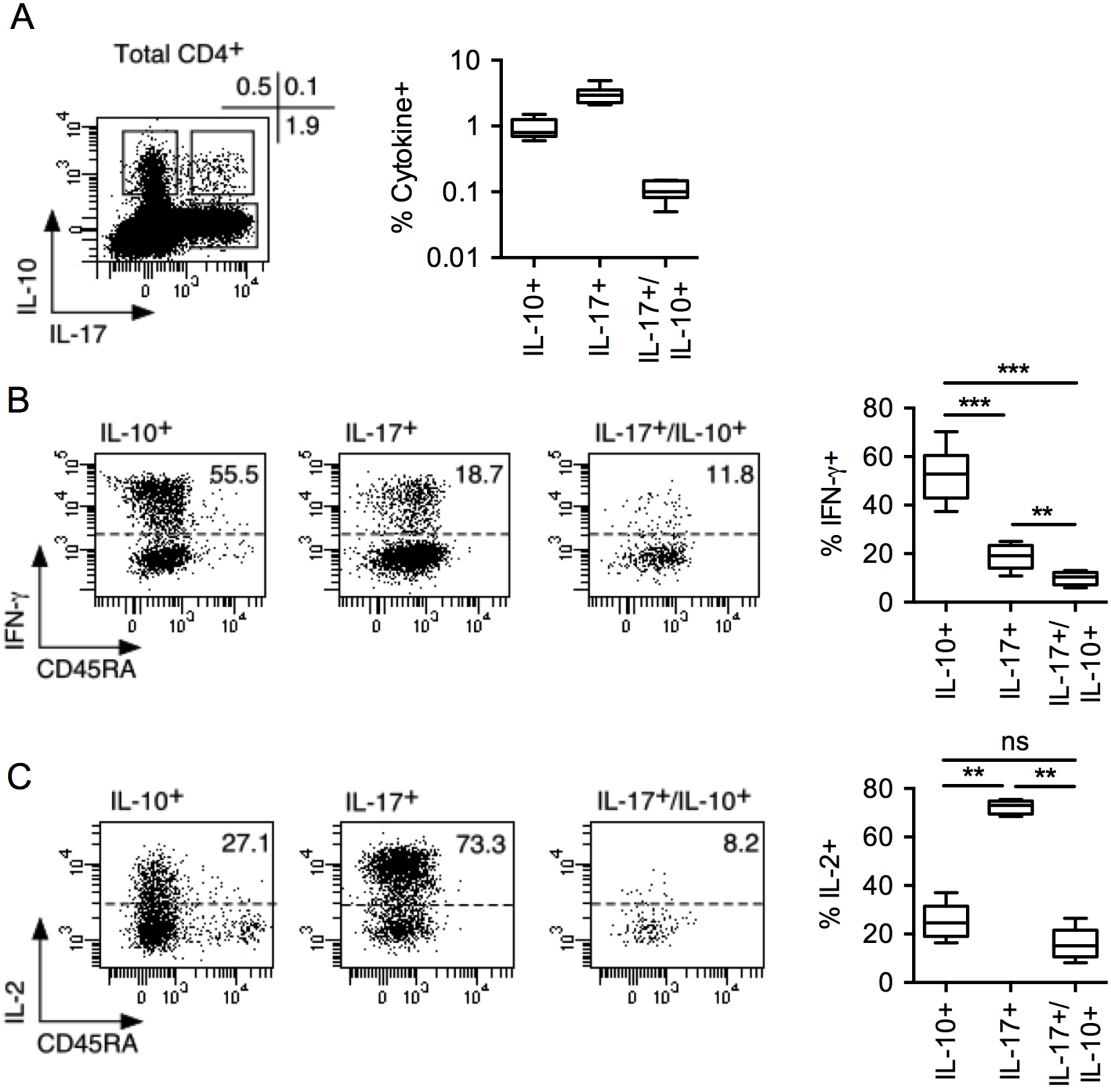
*Ex vivo* secretion of IL-10 and IL-17 in human circulating CD4 memory T cells. (A) *Ex vivo*–isolated CD4^+^ T cells were stimulated with PMA/ionomycin during 6hrs, stained with anti-CD45RA, -IL-10, -IL-17, -IFN-γ and -IL-2 mAbs and analyzed by flow cytometry. A dot plot, gated on total memory (CD45RA^−^) cells, is shown for one donor, and the proportions of memory CD4^+^ T cell secreting IL-10 or/and IL-17 *ex vivo* are summarized for all donors (*n* = 8). (B-C) Dot plots illustrating the co-secretion of IFN-γ and IL-2 in the indicated subpopulations are shown for one donor and the proportion of IFN-γ (*n* = 8) and IL-2 (*n* = 6) co-secreting cells in the indicated subpopulations is summarized. Statistical analyses were performed using the Mann—Whitney *U* test. **p < 0.01, ***p < 0.001, ns, Non-significant.

We initially assessed IL-10 and IL-17 secretion in circulating memory CD4 T cells from peripheral blood of healthy individuals stimulated *ex vivo* with PMA/ionomycin, by intracellular staining, using cytokine specific antibodies. We found that, in average, 0.9 ± 0.3% and 3 ± 0.9% of memory CD4 T cells secreted IL-10 or IL-17, respectively (Fig 1A). The majority of cells secreting IL-10 or IL-17 *ex vivo* appeared as distinct (i.e. for the majority of the cells the two cytokines were not co-secreted by the same cell). However, a subpopulation, representing about 0.1% of the cells, co-secreted the two cytokines. Different populations of IL-10 and IL-17 secreting CD4 T cells, that co-secrete or not IFN-γ, and exhibit distinct immune properties have been described [11]. We found that the IL-10 single secreting population contained high proportions of cells co-secreting IFN-γ (Fig 1B). In contrast, the IL-17 single secreting and, to an even higher extent, the IL-10/IL-17 co-secreting populations contained low proportions of cells secreting IFN-γ. In addition, whereas the majority of IL-17 single secreting cells co-secreted IL-2 *ex vivo* (Fig 1C), both the IL-10 single secreting and the IL-10/IL-17 co-secreting populations contained low proportions of cells secreting IL-2. This suggested that a significant proportion of cells within these populations could be anergic, i.e. unable to proliferate in the absence of exogenous IL-2, a characteristic that was reminiscent of FOXP3^+^ Treg.

### CD4 T cells secreting IL-10 *ex vivo*, alone or with IL-17, include FOXP3^-^ and FOXP3^+^ Helios^-^ Treg

**Fig 2:**
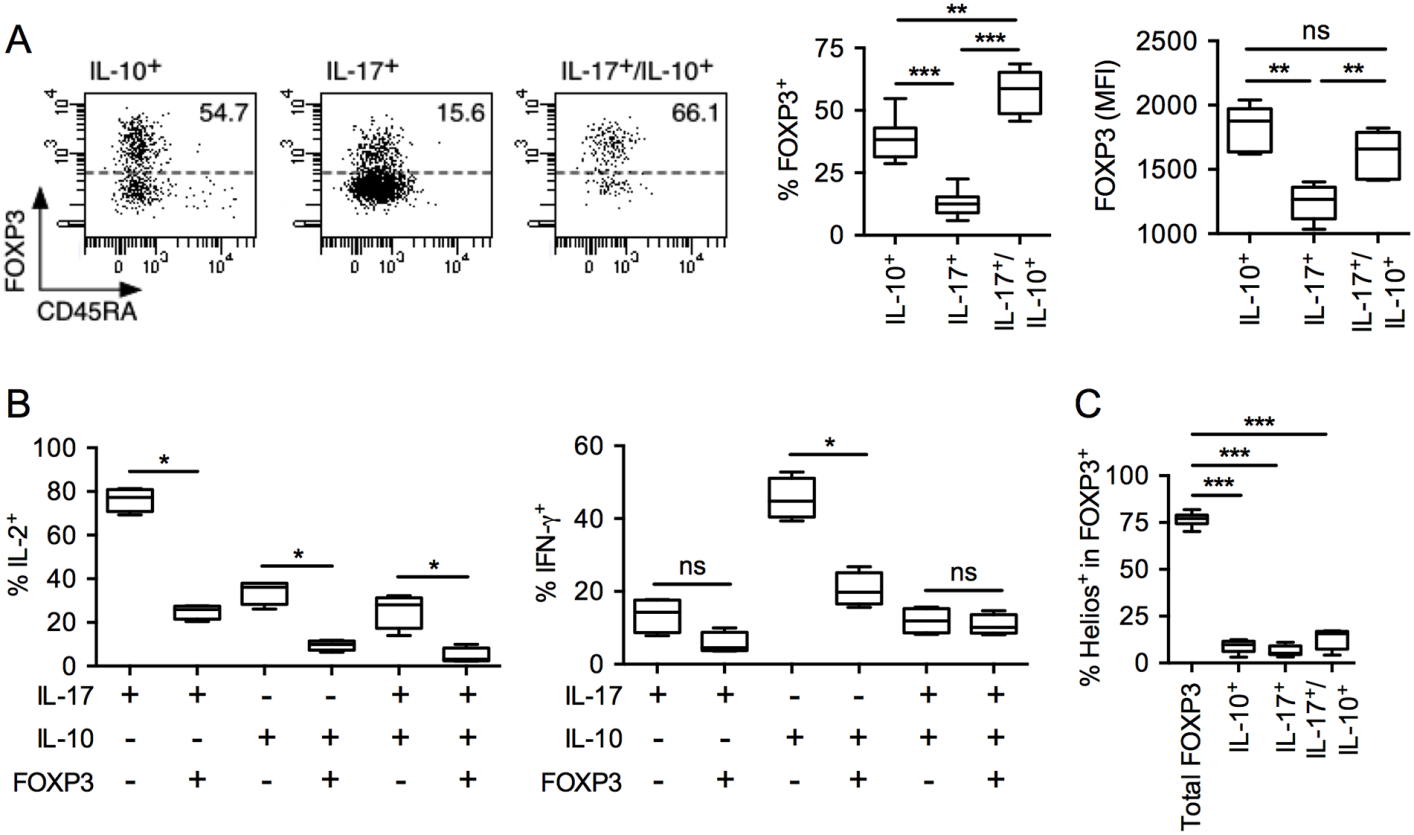
Circulating CD4 T cells secreting IL-10 *ex vivo*, alone or with IL-17, include FOXP3^-^ cells and FOXP3^+^ Helios^-^ pTreg. *Ex vivo*–isolated CD4^+^ T cells were stimulated with PMA/ionomycin, stained with anti-CD45RA, -IL-10, -IL-17, -FOXP3, and -Helios mAbs and analyzed by flow cytometry. (A) Dot plots depicting FOXP3 expressing cells within cytokine secreting populations are shown for one donor. The proportion of FOXP3 expressing cells within each cytokine secreting populations and their mean fluorescence intensity (MFI) are summarized for all donors (*n* = 8). (B) The proportions of cells co-secreting IL-2 or IFN-γ within the indicated populations are summarized for all donors (*n* = 4). (C) The proportions of Helios expressing cells within FOXP3^+^ Treg in the indicated populations are summarized for all donors (*n* = 8). Statistical analyses were performed using the Mann—Whitney *U* test. *p < 0.05, **p < 0.01, *** *p* < 0.001, ns, Non-significant.

Because of the above results and because we have previously described subpopulations of FOXP3^+^ Treg that produce IL-10 or IL-17 [3, 6], we analyzed the cytokine secreting populations with respect to expression of FOXP3. We found that each of the cytokine secreting subpopulations contained a sizable fraction of FOXP3^+^ cells (Fig 2A). The proportion of FOXP3^+^ cells, however, was significantly higher among IL-10 single secreting (39 ± 8%) than IL-17 single secreting (13 ± 5%) cells and represented 58 ± 9%, in average, of the cells co-secreting the two cytokines *ex vivo*. FOXP3 expression levels were variable among the populations and were higher among IL-10 single secreting and IL-10/IL-17 co-secreting cells as compared to IL-17 single secreting cells. As expected, in each population, IL-2 secretion was confined to cells expressing low levels of FOXP3 (Fig 2B). In addition, for the IL-10 secreting population IFN-γ secreting cells were also significantly more frequent in FOXP3^-^ than in FOXP3^+^ cells. It is noteworthy that the majority of FOXP3^+^ Treg within each of the cytokine secreting populations failed to express the transcription factor Helios, that is expressed by thymically derived FOXP3^+^ Treg (tTreg), suggesting that these cells are peripherally induced (pTreg) rather than tTreg (Fig 2C). In contrast, no significant secretion of cytokines was detected in the Helios^+^ Treg population (not shown).

### IL-10 and IL-17 secretion levels vary depending on the mode of stimulation

**Fig 3:**
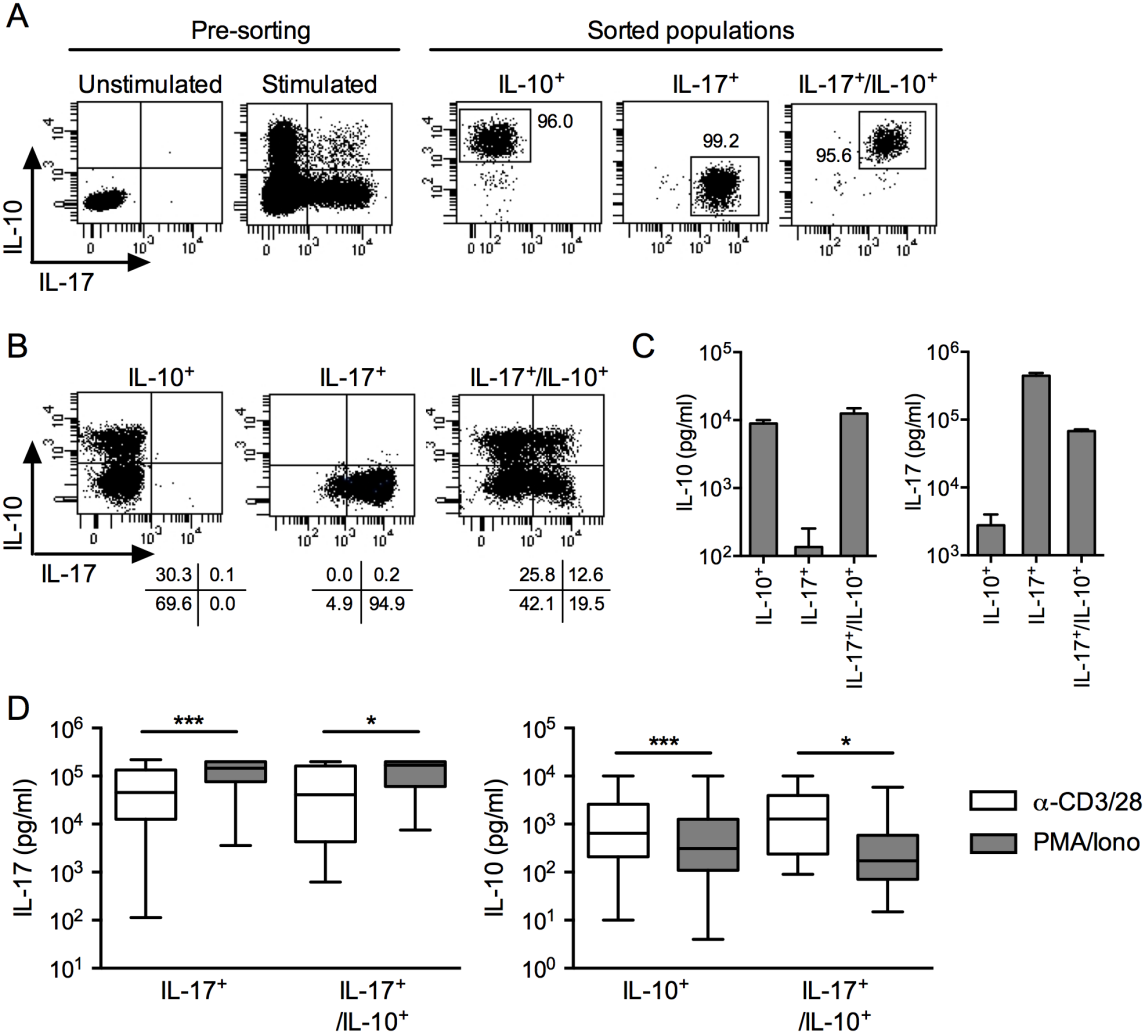
*Ex vivo* isolation of IL-10 or/and IL-17 secreting populations and generation of clones. (A) CD4^+^ T cells were stimulated *ex vivo* with PMA/ionomycin. Cells secreting IL-10 and/or IL-17 were assessed using a two-color cytokine secretion assay combining the IL-10 and the IL-17 secretion assays from Miltenyi Biotec and sorted by flow cytometry to a high degree of purity. Dot plots show the cytokine secreting populations prior to and after cell sorting. (B-C) Clones were generated by limiting dilution culture and assessed for cytokine secretion after PMA/ionomycin stimulation by intracellular staining or by ELISA. (B) Dot plots are shown for representative IL-10^+^, IL-17^+^ and IL-17^+^/IL-10^+^ clones. (C) The levels of IL-10 and IL-17 secreted by representative clone of each population were assessed in the 24hrs culture supernatants after PMA/ionomycin stimulation by ELISA. Mean and SEM of duplicates are shown. (D) IL-17^+^ clones (*n* = 136), IL-10^+^ clones (*n* = 275) and IL-17^+^/IL-10^+^ clones (*n* = 17) were assessed for cytokine secretion by ELISA. Data are representative of three independent experiments. Statistical analyses were performed using the Mann—Whitney *U* test. *p < 0.05, ***p < 0.001.

**Fig 4:**
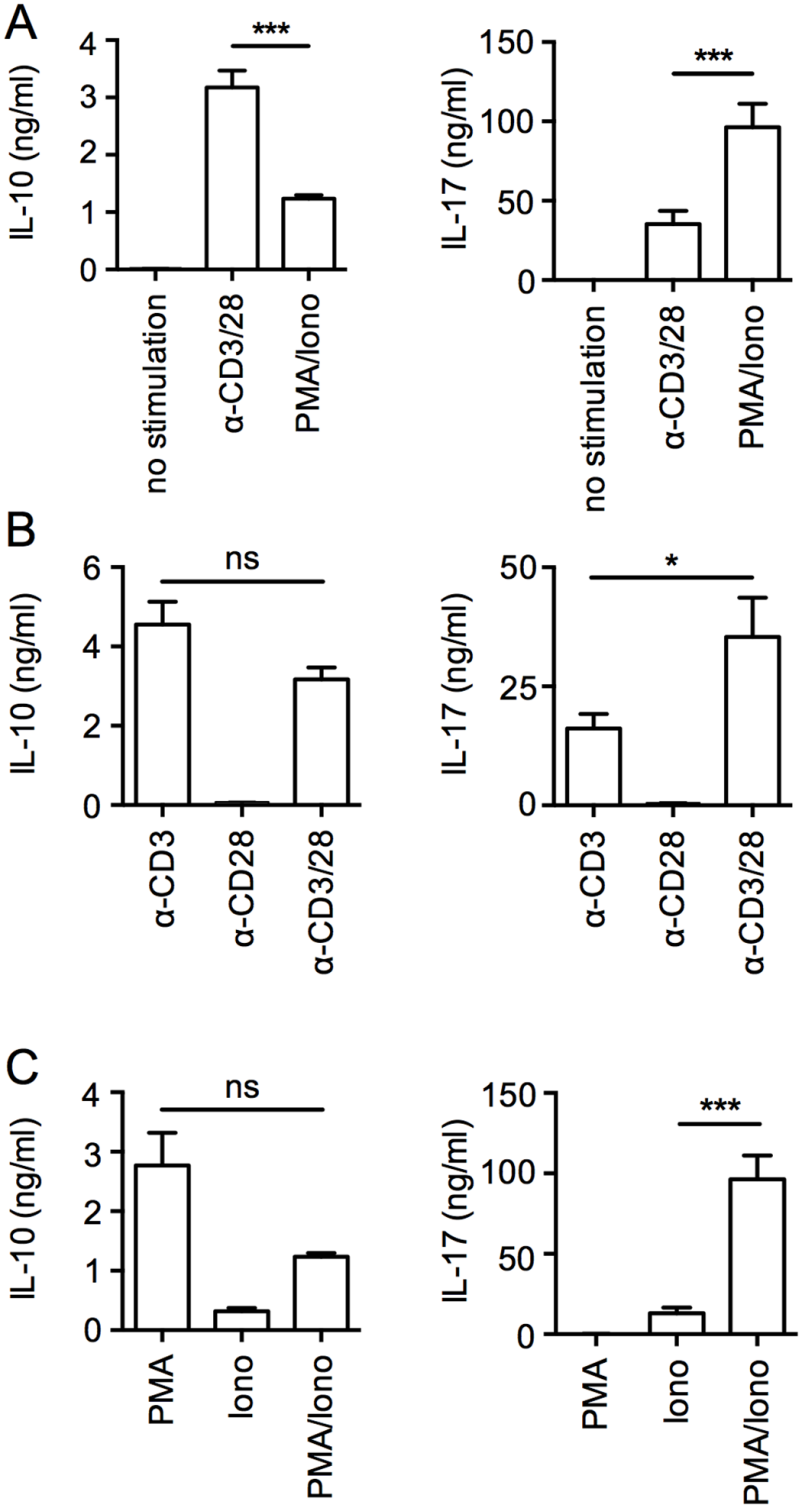
IL-10 and IL-17 secretion levels of clonal CD4 T cells vary depending on the mode of stimulation. Pools of IL-10^+^ clones and IL-17^+^/IL-10^+^ clones (left side) or pools of IL-17^+^ clones and IL-17^+^/IL-10^+^ clones (right side) were stimulated with anti-CD3 and anti-CD28 or with PMA and ionomycin (A), or with anti-CD3 and anti-CD28, alone or in combination (B), or with PMA and ionomycin, alone or in combination (C). The levels of IL-10 and IL-17 secreted were assessed in the 24hrs culture supernatants, by ELISA. Data shown are mean with SEM of duplicates and representative of four independent experiments. Statistical analyses were performed using the Mann—Whitney *U* test. *p < 0.05, ***p < 0.001, ns, Non-significant.

To further assess IL-10 or/and IL-17 secreting populations functionally, we isolated *ex vivo* IL-10 single secreting, IL-17 single secreting and IL-10/IL-17 double secreting populations using the Miltenyi secretion/detection assay and flow cytometry cell sorting. As illustrated in Fig 3A, populations with the selected cytokine secretion profile were isolated to a high level of purity (>95%). We stimulated the isolated cells with PHA and feeder cells under limiting dilution conditions to generate clones that we reassessed for cytokines secretion, by intracellular staining and ELISA (Fig 3B and 3C). We then compared cytokine production by the clones after stimulation with anti-CD3/CD28 antibodies or PMA/ionomycin (Fig 3D). We detected significantly higher levels of IL-17 following stimulation with PMA/ionomycin as compared to anti-CD3/28 antibodies. The opposite was true for IL-10 that was prevalently secreted following stimulation with anti-CD3/28 antibodies rather than with PMA/ionomycin (Figs 3D and 4A). Thus, the levels of secretion of IL-10 and IL-17 varied significantly depending on the mode of stimulation, namely by activating the cells through TCR-mediated stimulation using anti-CD3/CD28 antibodies versus using PMA plus ionomycin, a combination that activates the cells independently of TCR, through the Ca2^+^/calmodulin-dependent and the PKC signaling pathways. To further explore the relative contribution of these stimuli to the secretion of the cytokines, we stimulated IL-10/IL-17 secreting cells with each of them, separately or in combination. We found that stimulation with anti-CD3 alone was optimal for inducing IL-10 secretion, whereas the addition of anti-CD28 did not increase the response (Fig 4B *)*. In contrast, IL-17 secretion was low following stimulation with anti-CD3 antibodies and was significantly increased in the presence of anti-CD28 antibodies. In addition, we found that secretion of IL-10 following stimulation with PMA was not increased by addition of ionomycin, whereas secretion of IL-17 required the simultaneous addition of PMA and ionomycin (Fig 4C).

### IL-10 and IL-17 secretion levels vary depending on the time after stimulation

**Fig 5:**
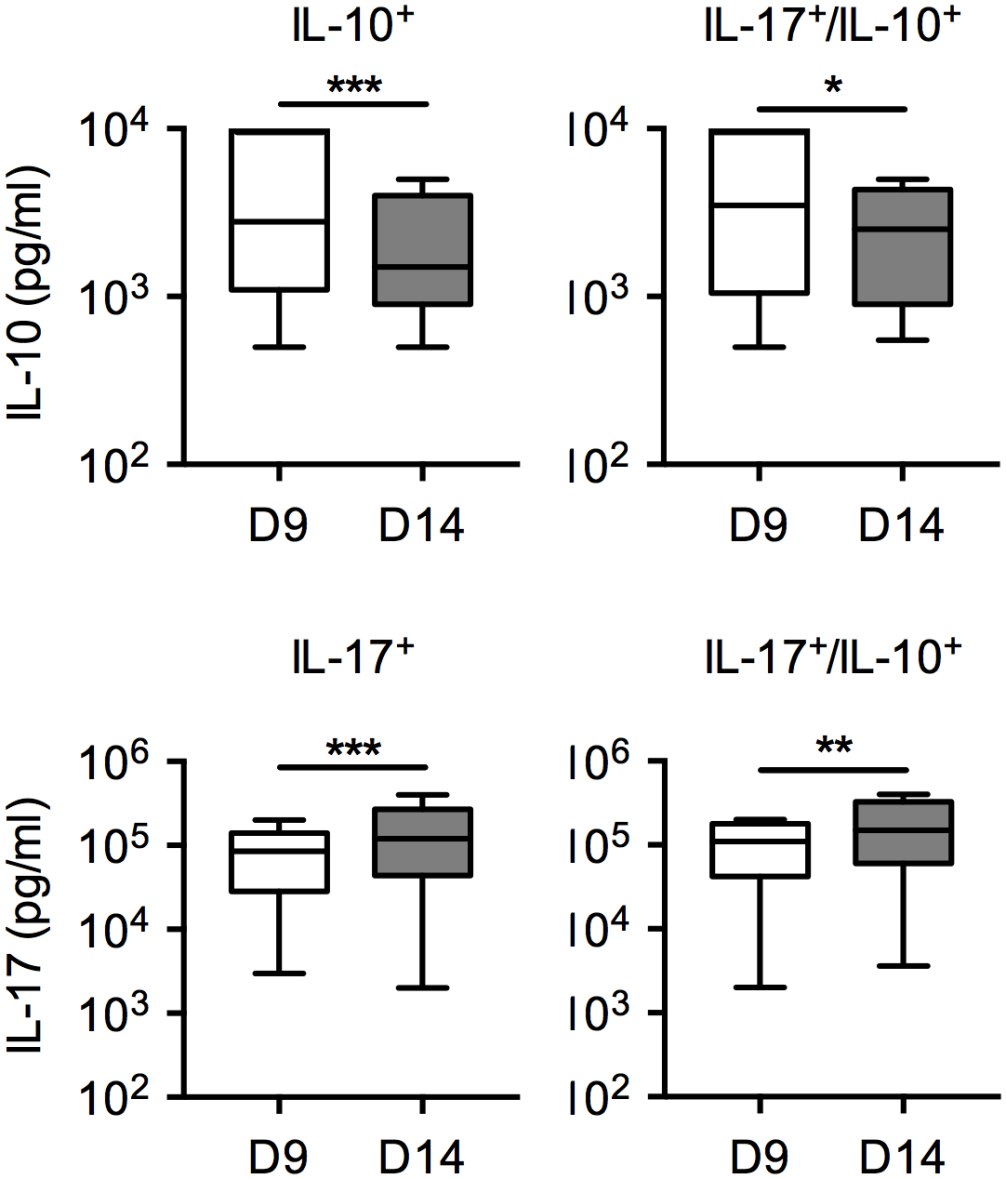
IL-10 and IL-17 secretion levels of clonal CD4 T cells vary depending on time after stimulation. IL-10^+^ clones (n = 137), IL-17^+^ clones (n = 144) and IL-17^+^/IL-10^+^ clones (n = 83) were stimulated with anti-CD3/28, rested for 9 or 14 days, then restimulated. The levels of IL-10 and IL-17 secreted were assessed in the 24hrs culture supernatants, after stimulation, by ELISA. Statistical analyses were performed using the Mann—Whitney *U* test. *p <0.05, **p<0.01, ***p < 0.001.

It has been recently reported that, for T_H_17 clones specific for Staphylococcus Aureus that co-secrete IL-10 and IL-17, the levels of secreted cytokines vary significantly depending on time after stimulation, being higher for IL-10 in the early days after stimulation, when the cells are in an activated state, and for IL-17 at later time points, when they are in a resting state [11]. To address if this was also true for the populations under analysis in this study, we compared the cytokine secretion capacity of the clones at day 9 and 14 after stimulation. We found that for both IL-17/IL-10 double secreting clones and IL-10 single secreting clones IL-10 secretion was indeed significantly higher at day 9 than at day 14 after stimulation (Fig 5). The opposite was true for IL-17 that was secreted at higher levels at day 14 than at day 9 after stimulation.

### Secretion of IL-10 but not of IL-17 is increased in the presence of sodium butyrate

**Fig 6:**
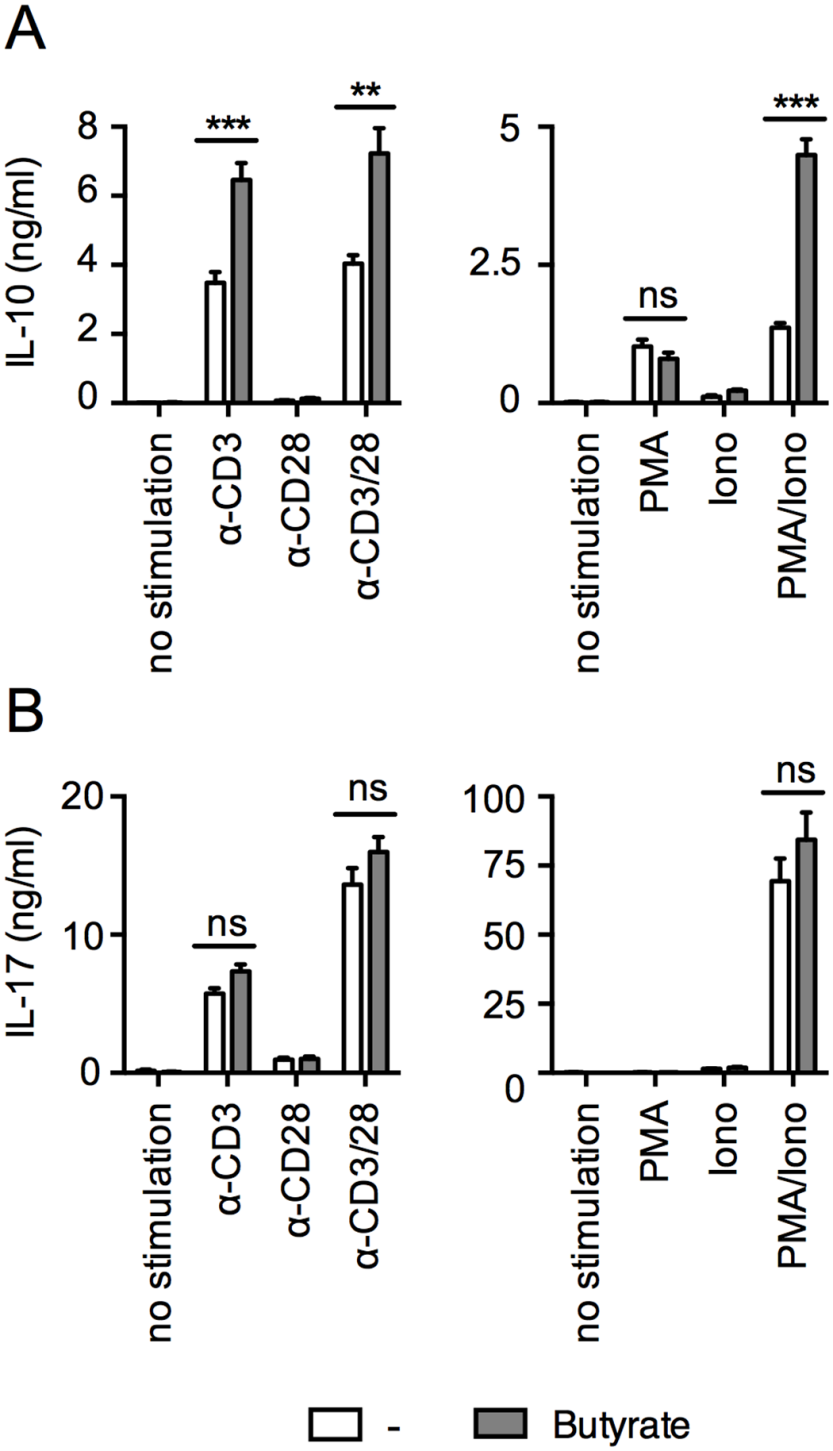
Secretion of IL-10 but not of IL-17 is increased in the presence of sodium butyrate. Pools of IL-10^+^ clones and IL-17^+^/IL-10^+^ clones (A) or pools of IL-17^+^ clones and IL-17^+^/IL-10^+^ clones (B) were stimulated with anti-CD3 and anti-CD28 alone or in combination or with PMA and ionomycin, alone or in combination, in the absence or in the presence of sodium butyrate. The levels of IL-10 (A) and IL-17 (B) secreted were assessed in the 24hrs culture supernatants, by ELISA. Data are shown as mean with SEM of duplicates. Data are representative of three independent experiments. Statistical analyses were performed using the Mann—Whitney *U* test. **p < 0.01, ***p < 0.001, ns, Non-significant.

The capacity of IL-10 and IL-17 secreting cells to increase IL-10 secretion and decrease IL-17 secretion in response to persistent stimulation, a situation that occurs in the case of immune responses to commensal organisms in the gut, prompted us to assess if metabolites produced by commensal bacteria could participate in maintaining a prevalent IL-10 secreting tolerogenic profile in these cells. To address this hypothesis, we stimulated the clones in the absence or in the presence of sodium butyrate, a short chain fatty acid produced by bacterial fermentation that is involved in controlling gut inflammatory responses [12, 13]. As illustrated in Fig 6A, we found that stimulation in the presence of sodium butyrate with either anti-CD3 alone or together with anti-CD28 or with PMA/ionomycin resulted in a significant increase in IL-10 secretion. In contrast, the presence of butyrate did not significantly alter the secretion levels of IL-17 (Fig 6B).

## Discussion

The cytokines IL-17 and IL-10 exert opposite immune functions. IL-17 is pro-inflammatory and recruits monocytes and neutrophils at the site of inflammation by inducing the production of chemokines and other cytokines [14]. IL-10, in contrast, is a potently immunosuppressive and anti-inflammatory cytokine, that down-regulates the production of type I cytokines and the expression of MHC class II and co-stimulatory molecules on antigen presenting cells [15]. CD4 T cells that secrete the two cytokines are abundant in the gut, where their equilibrium contributes to regulate tolerance and immunity to commensal microorganisms that represent a large and constantly present source of foreign antigens [16, 17]. CD4 T cells secreting IL-17 or IL-10 include T_H_17 cells, Tr1 cells and FOXP3^+^ Treg. These populations, both conventional and Treg, have been considered to be mostly distinct. However, several studies have suggested a close relationship between some of them. For example, T_H_17 and FOXP3^+^ iTreg both require TGF-β for their differentiation, and suppressive FOXP3^+^ Treg that secrete IL-17 have been described [6]. In addition, ablation of a conserved non-coding element within the FOXP3 locus that recruits Smad in response to TGF-β impaired iTreg generation and increased the levels of Tr1, surprisingly without affecting those of T_H_17 [18], suggesting that Tr1 and iTreg may also represent alternative fates of CD4 T cell differentiation. Therefore, overall, the relationship between T_H_17, Tr1 and iTreg, in terms of differentiation, specificity, function and plasticity remains incompletely understood. Adding to this complexity, microbe specific CD4 T cells that co-secrete IL-17 and IL-10 have been recently described [11]. However, the prevalence, characteristics, mode of action and potential role of this type of populations have not been fully addressed.

To get insight into these questions, we assessed circulating CD4 T cells that secrete IL-17 and IL-10 *ex vivo*, separately or in association. Whereas most CD4 T cells secreting IL-10 or IL-17 *ex vivo* appeared as distinct (i.e. IL-10 and IL-17 were secreted by different cells), we identified a subpopulation of CD4 T cells that co-secrete the two cytokines. We found that, consistent with our previous identification of FOXP3^+^ Treg that secrete IL-17 *ex vivo* [6], about 15% of the cells that secreted IL-17 alone *ex vivo* were FOXP3^+^. In addition, about half of the cells secreting IL-10 alone and an even larger proportion (>60%) of those that co-secreted IL-10 and IL-17 were FOXP3^+^. These FOXP3^+^ Treg failed to express the transcription factor Helios [19], of the Ikaros family, suggesting that they mostly represent peripherally induced rather than natural (i.e. thymically derived) Treg. These results underline the heterogeneity of human pTreg, and further support their close relationship with T_H_17 and Tr1 type cells. Using a cell enrichment assay based on cytokine secretion followed by *in vitro* expansion under limiting dilution conditions, we isolated clonal CD4 T cell populations secreting IL-10 or/and IL-17 and determined the conditions under which each cytokine is optimally secreted. We found that the capacity of IL-10/IL-17 co-secreting cells to preferentially secrete IL-10 or IL-17 was reciprocally regulated depending on the mode of stimulation. Namely, IL-10 was optimally secreted following TCR-mediated stimulation, independent of co-stimulation, whereas, conversely, IL-17 secretion was higher in the presence of costimulation and was optimal following activation of the PKC pathway that bypasses the TCR. Better secretion of IL-17 by TCR independent stimulation is compatible with the recently proposed concept that T cells secreting IL-17 have impaired TCR signaling due to reduced phosphorylation of the CD3ζ chain as well as of ZAP-70 and SLP-76, that are direct targets of the CD3ζ chain kinase activity [20]. The decreased sensitivity of T_H_17 cells (as compared to T_H_1) to TCR mediated stimulation has been proposed to be a self-regulatory mechanism that limits their pathogenicity [20]. Optimal secretion of IL-10 following TCR stimulation in the absence of co-stimulation is in line with the results from previous studies [21]. The finding that IL-10 is better produced after stimulation with anti-CD3/28 antibodies than with PMA/ionomycin, whereas the opposite is true for IL-17, has been reported by others [22]. Increased secretion of IL-10 upon TCR stimulation as compared to activation of the PKC pathway alone is likely to be related to the involvement of different signaling pathways in the secretion of IL-10 with respect to IL-17. One candidate is the MAPK P38 pathway that is involved in IL-10 secretion following TCR-mediated stimulation but is not activated by stimulation with PMA and ionomycin. The capacity to secrete one or the other cytokine was dependent on the time after stimulation, with IL-10 being optimally secreted early after stimulation and IL-17 at later time points, when the cells had reverted to a resting state. Downregulation of IL-17 production by microorganism specific T_H_17 cells in the early phases after stimulation has been reported by Zielinsky CE et al. and partially attributed to the dowregulation of the transcription factor RORγt, that controls IL-17 secretion [11]. On the other hand, the levels of secreted IL-10, but not of IL-17, were significantly increased in the presence of butyrate, a short chain fatty acid generated by bacterial fermentation of dietary fibers in the colon [23], underlying the role of locally produced metabolites on the cytokine secretion profile of resident populations.

Together, these results are compatible with the concept that, based on the differential molecular mechanisms that regulate the secretion of IL-10 and IL-17, CD4 T cells that are able to secrete both cytokines will secrete high levels of IL-10 when chronically stimulated by persistent antigens, such as those from microbiota, presented in the steady state by non professional APC, and enhanced in the presence of locally produced microbial metabolites. In contrast, the same cells will decrease IL-10 secretion and increase IL-17 secretion when they encounter the same microorganisms after barrier breaching, i.e. in the lamina propia or at distal sites. Together, our results contribute to elucidate how, because of the capacity of IL-10/IL-17 secreting cells to modulate their cytokine secretion profile depending on conditions, specific memory cells can simultaneously contribute to maintain tolerance to commensal microorganisms when they are present in their physiologic context, while maintaining the ability to protect us from the same microorganisms if they become potentially pathogenic at distal sites.

## References

[1] Haringer B, Lozza L, Steckel B, Geginat J. Identification and characterization of IL-10/IFN-gamma-producing effector-like T cells with regulatory function in human blood. J Exp Med. 2009;206(5):1009–17. 10.1084/jem.20082238 19414553 PMC2715038

[2] Pot C, Apetoh L, Kuchroo VK. Type 1 regulatory T cells (Tr1) in autoimmunity. Seminars in immunology. 2011;23(3):202–8. 21840222 10.1016/j.smim.2011.07.005 PMC3178065

[3] Raffin C, Pignon P, Celse C, Debien E, Valmori D, Ayyoub M. Human memory Helios- FOXP3+ regulatory T cells (Tregs) encompass induced Tregs that express Aiolos and respond to IL-1beta by downregulating their suppressor functions. J Immunol. 2013;191(9):4619–27. 10.4049/jimmunol.1301378 . 24068664

[4] Hamai A, Pignon P, Raimbaud I, Duperrier-Amouriaux K, Senellart H, Hiret S, et al Human T(H)17 immune cells specific for the tumor antigen MAGE-A3 convert to IFN-gamma-secreting cells as they differentiate into effector T cells in vivo. Cancer Res. 2012;72(5):1059–63. 10.1158/0008-5472.CAN-11-3432 . 22253231

[5] Zelante T, De Luca A, D'Angelo C, Moretti S, Romani L. IL-17/Th17 in anti-fungal immunity: what's new? European journal of immunology. 2009;39(3):645–8. Epub 2009/03/14. 10.1002/eji.200839102 . 19283705

[6] Ayyoub M, Deknuydt F, Raimbaud I, Dousset C, Leveque L, Bioley G, et al Human memory FOXP3+ Tregs secrete IL-17 ex vivo and constitutively express the T(H)17 lineage-specific transcription factor RORgamma t. Proceedings of the National Academy of Sciences of the United States of America. 2009;106(21):8635–40. Epub 2009/05/15. 10.1073/pnas.0900621106 ; PubMed Central PMCID: PMCPmc2688993. 19439651 PMC2688993

[7] Voo KS, Wang YH, Santori FR, Boggiano C, Wang YH, Arima K, et al Identification of IL-17-producing FOXP3+ regulatory T cells in humans. Proc Natl Acad Sci U S A. 2009;106(12):4793–8. 10.1073/pnas.0900408106 19273860 PMC2653560

[8] Chalmin F, Mignot G, Bruchard M, Chevriaux A, Vegran F, Hichami A, et al Stat3 and Gfi-1 transcription factors control Th17 cell immunosuppressive activity via the regulation of ectonucleotidase expression. Immunity. 2012;36(3):362–73. 10.1016/j.immuni.2011.12.019 . 22406269

[9] Nutsch KM, Hsieh CS. T cell tolerance and immunity to commensal bacteria. Current opinion in immunology. 2012;24(4):385–91. Epub 2012/05/23. 10.1016/j.coi.2012.04.009 ; PubMed Central PMCID: PMCPmc3423487. 22613090 PMC3423487

[10] Littman DR, Rudensky AY. Th17 and regulatory T cells in mediating and restraining inflammation. Cell. 2010;140(6):845–58. 10.1016/j.cell.2010.02.021 . 20303875

[11] Zielinski CE, Mele F, Aschenbrenner D, Jarrossay D, Ronchi F, Gattorno M, et al Pathogen-induced human TH17 cells produce IFN-gamma or IL-10 and are regulated by IL-1beta. Nature. 2012;484(7395):514–8. 10.1038/nature10957 . 22466287

[12] Arpaia N, Rudensky AY. Microbial metabolites control gut inflammatory responses. Proc Natl Acad Sci U S A. 2014;111(6):2058–9. 10.1073/pnas.1323183111 24434557 PMC3926042

[13] Furusawa Y, Obata Y, Fukuda S, Endo TA, Nakato G, Takahashi D, et al Commensal microbe-derived butyrate induces the differentiation of colonic regulatory T cells. Nature. 2013;504(7480):446–50. Epub 2013/11/15. 10.1038/nature12721 . 24226770

[14] Weaver CT, Hatton RD, Mangan PR, Harrington LE. IL-17 family cytokines and the expanding diversity of effector T cell lineages. Annual review of immunology. 2007;25:821–52. 10.1146/annurev.immunol.25.022106.141557 . 17201677

[15] Sabat R, Grutz G, Warszawska K, Kirsch S, Witte E, Wolk K, et al Biology of interleukin-10. Cytokine & growth factor reviews. 2010;21(5):331–44. 10.1016/j.cytogfr.2010.09.002 . 21115385

[16] Kole A, Maloy KJ. Control of intestinal inflammation by interleukin-10. Current topics in microbiology and immunology. 2014;380:19–38. 10.1007/978-3-662-43492-5_2 . 25004812

[17] Blaschitz C, Raffatellu M. Th17 cytokines and the gut mucosal barrier. Journal of clinical immunology. 2010;30(2):196–203. 10.1007/s10875-010-9368-7 20127275 PMC2842875

[18] Zheng Y, Josefowicz S, Chaudhry A, Peng XP, Forbush K, Rudensky AY. Role of conserved non-coding DNA elements in the Foxp3 gene in regulatory T-cell fate. Nature. 2010;463(7282):808–12. 10.1038/nature08750 20072126 PMC2884187

[19] Thornton AM, Korty PE, Tran DQ, Wohlfert EA, Murray PE, Belkaid Y, et al Expression of Helios, an Ikaros transcription factor family member, differentiates thymic-derived from peripherally induced Foxp3+ T regulatory cells. J Immunol. 2010;184(7):3433–41. 10.4049/jimmunol.0904028 20181882 PMC3725574

[20] Santarlasci V, Maggi L, Capone M, Querci V, Beltrame L, Cavalieri D, et al Rarity of human T helper 17 cells is due to retinoic acid orphan receptor-dependent mechanisms that limit their expansion. Immunity. 2012;36(2):201–14. 10.1016/j.immuni.2011.12.013 . 22326581

[21] Rivino L, Gruarin P, Haringer B, Steinfelder S, Lozza L, Steckel B, et al CCR6 is expressed on an IL-10-producing, autoreactive memory T cell population with context-dependent regulatory function. The Journal of experimental medicine. 2010;207(3):565–77. Epub 2010/03/03. 10.1084/jem.20091021 ; PubMed Central PMCID: PMCPmc2839148. 20194631 PMC2839148

[22] Olsen I, Sollid LM. Pitfalls in determining the cytokine profile of human T cells. Journal of immunological methods. 2013;390(1–2):106–12. 10.1016/j.jim.2013.01.015 . 23416458

[23] Ganapathy V, Thangaraju M, Prasad PD, Martin PM, Singh N. Transporters and receptors for short-chain fatty acids as the molecular link between colonic bacteria and the host. Current opinion in pharmacology. 2013;13(6):869–74. 10.1016/j.coph.2013.08.006 . 23978504

